# Controllable Fabrication of Molecularly Imprinted Microspheres with Nanoporous and Multilayered Structure for Dialysate Regeneration

**DOI:** 10.3390/nano12030418

**Published:** 2022-01-27

**Authors:** Hongchi Wu, Shanguo Zhang, Lu Liu, Yukun Ren, Chun Xue, Wenlong Wu, Xiaoming Chen, Hongyuan Jiang

**Affiliations:** 1 Department of Nephrology, First Affiliated Hospital of Harbin Medical University, 23 Youzheng Street, Harbin 150001, China; 2020020649@hrbmu.edu.cn (L.L.); 2021020689@hrbmu.edu.cn (C.X.); 2 School of Mechatronics Engineering, Harbin Institute of Technology, West Da-zhi Street 92, Harbin 150001, China; 21b908081@stu.hit.edu.cn (S.Z.); rykhit@hit.edu.cn (Y.R.); hit_wu@stu.hit.edu.cn (W.W.); 3 State Key Laboratory of Robotics and System, Harbin Institute of Technology, West Da-zhi Street 92, Harbin 150001, China; 4 School of Control Engineering, Northeastern University at Qinhuangdao, Qinhuangdao 066004, China; chenxiaoming@neuq.edu.cn

**Keywords:** liquid–liquid phase separation, nanoporous microspheres, microfluidic technology, urea absorption

## Abstract

Adsorption of urea from dialysate is essential for wearable artificial kidneys (WRK). Molecularly imprinted microspheres with nanoporous and multilayered structures are prepared based on liquid–liquid phase separation (LLPS), which can selectively adsorb urea. In addition, we combine the microspheres with a designed polydimethylsiloxane (PDMS) chip to propose an efficient urea adsorption platform. In this work, we propose a formulation of LLPS including Tripropylene glycol diacrylate (TPGDA), ethanol, and acrylic acid (30% *v*/*v*), to prepare urea molecularly imprinted microspheres in a simple and highly controllable method. These microspheres have urea molecular imprinting sites on the surface and inside, allowing selective adsorption of urea and preservation of other essential constituents. Previous static studies on urea adsorption have not considered the combination between urea adsorbent and WRK. Therefore, we design the platform embedded with urea molecular imprinted microspheres, which can disturb the fluid motion and improve the efficiency of urea adsorption. These advantages enable the urea absorption platform to be highly promising for dialysate regeneration in WRK.

## 1. Introduction

Chronic renal failure refers to chronic progressive renal parenchymal damage caused by various factors, resulting in irreversible atrophy of the kidneys [1]. The clinical symptoms of this disease mainly include retention of metabolites in the body, imbalance of water, electrolytes, and acid–base balance [2]. According to statistics, the number of people suffering from chronic kidney disease worldwide was nearly 700 million in 2017, with 1.2 million deaths. Besides, the number of deaths is expected to rise to 2 million (even 4 million) by 2040 [3]. When chronic kidney disease progresses to end-stage renal disease (ESRD), the glomerular filtration rate is unable to meet the body’s physiological needs. Thus, dialysis is the main way to keep most patients alive for the rest of their lives apart from expensive kidney transplants. However, traditional dialysis requires patients to acquire treatment in a special hospital at least three times a week for about four hours each time, which severely affects the normal life of the patient. Dialysate is a chemical bath used by uremic patients during dialysis to draw fluid and toxins from the bloodstream and to supply electrolytes and other beneficial chemicals to the body [4]. In the process, approximately 120 L of dialysate is used. The portable wearable artificial kidney (WRK) can imitate the human kidney for continuous dialysis, which can greatly improve the quality of life of patients [5,6]. Continuous dialysis of WRK requires a small volume and a sufficient supply of dialysate [7,8]. One of the important research areas in the development of WRK is to regenerate dialysate in a closed-loop system. Since urea is much more abundant in dialysate than other waste solutes and is difficult to remove, an important obstacle for dialysate regeneration is the removal of urea [9].

At present, scholars have proposed a series of strategies to remove urea from the dialysate. Urea can be converted to gas by electrochemical method, but toxic substances such as chloramines may be generated at the same time [10]. Activated carbon can adsorb urea but has a poor affinity with urea [11,12]. Chitosan-based metal ion complexes have a high urea adsorption capacity, but the potential release of metal ions increases the toxicity of the adsorbent [13]. However, most of the studies above investigated the adsorption capacity of urea adsorbent in containers (e.g., test tubes and beakers), and less attention was given to the method of combining urea adsorbent with WRK. Besides, these studies focused on the urea adsorption capacity of the adsorbent and neglected the effect on the beneficial substances in the dialysate. Therefore, it is necessary to investigate a specific urea adsorption platform for dialysate regeneration in the development of WRK.

**Figure 1:**
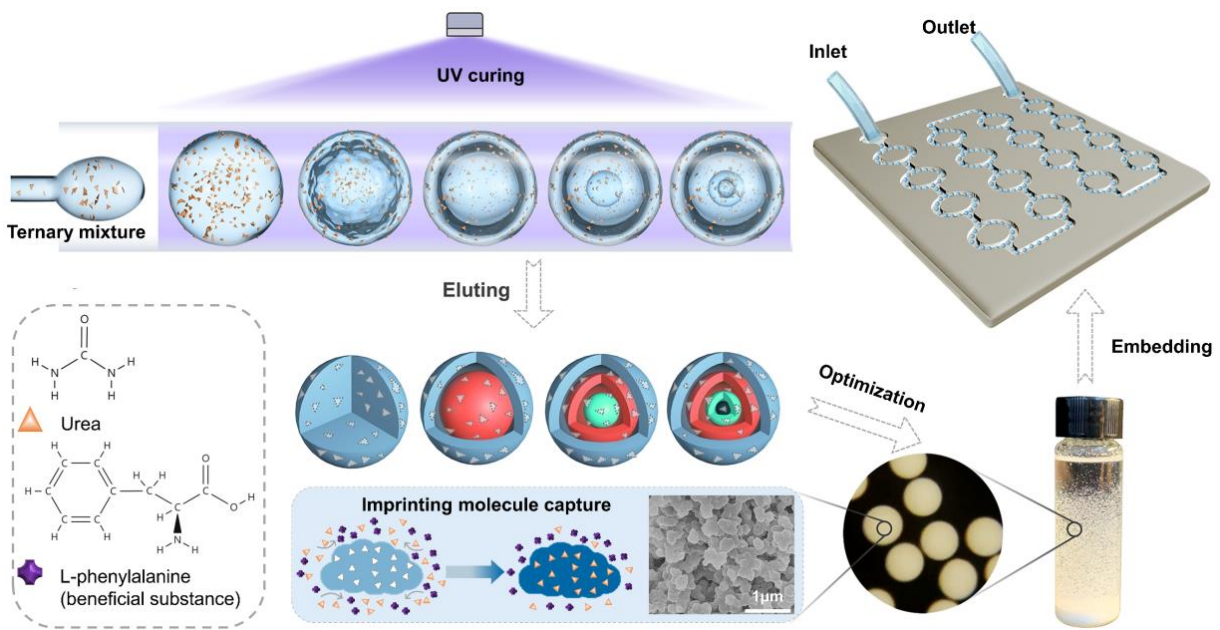
Schematic illustration of the fabrication processes of the nanoporous MISP with multi-layered structures and the mechanism of the MISP integrated microfluidic chip for urea absorption.

In this paper, we proposed a urea adsorption platform with molecularly imprinted nanoporous microspheres with multi-layered structures embedded in the channel, as shown in Figure 1. First, we developed a new mass-transfer-induced ternary liquid–liquid phase separation (LLSP) agent system including TPGDA, ethanol, 30% *v*/*v* of acrylic acid aqueous solution (TAA) to prepare multi-emulsion droplets. We studied the droplet phase separation regular of this ternary system in the out phases of poly (vinyl alcohol) (PVA) solution and silicone oil, respectively. Then, based on this LLSP, urea molecularly imprinted microspheres (MISP) were prepared, which were composites of TPGDA and polyacrylic acid (PAA). The TPGDA and PAA formed after curing are biocompatible materials and can be used for biomedical applications [14,15,16]. Afterward, the chemical bonding changes and thermal stability of MISP were analyzed by comparing them with TPGDA and PAA. Next, we investigated the adsorption kinetic properties, isothermal adsorption properties, and selective adsorption properties of MISP and non-molecularly imprinted microspheres (NISP). The results showed that MISP had a property of special adsorption for urea than other beneficial materials. Finally, the MISP was embedded in the designed microfluidic platform, and the adsorption efficiency was significantly improved due to the enhanced contact between the fluid and the MISP microsphere. Meanwhile, we verified the perturbation process of the fluid in the platform using numerical simulations. Thus, this platform presents good potential in the development of WRK.

## 2. Materials and Methods

### 2.1. Materials

Tripropylene Glycol Diacrylate (TPGDA, 90% of the total of isomer, stabilized with MEHQ), urea, poly(vinyl alcohol) (PVA, *M*w = 13,000–23,000), and dimethylaminobenzaldehyde (DMAB), were purchased from Aladdin Bio-Chem Technology Co., Ltd. (Shanghai, China). The silicone oil (Dow Corning, viscosity~50 mPa·s) was obtained from CChip scientific instrument Co., Ltd. (Suzhou, China). Rhodamine B and 2-hydroxy-2-methylpropiophenone (HMPP) were obtained from Sigma-Aldrich Trading Co., Ltd. (Shanghai, China). Acrylic acid (AA) was purchased from Macklin Biochemical Co.,Ltd. (Shanghai, China). Phosphate buffer saline (PBS, 0.1 M) was obtained from Hewei Pharmaceutical Technology Co., Ltd. (Guangzhou, China). Absolute ethanol was obtained from Damao chemical reagent factory (Tianjin, China).

### 2.2. Formation of Multi-Emulsion Droplets

The formation of initial single-emulsion droplets was carried out in a coaxial capillary microfluidic device. The method of fabricating the microfluidic device can refer to our previous reports [17]. In detail, we prepared a conventional co-flow glass capillary microfluidic chip to generate the initial droplets for liquid–liquid phase separation, with 900 μm and 60 μm inner diameters for continuous and disperse phase glass capillaries, respectively. The continuous phase liquid and the dispersed phase liquid were injected into the corresponding glass capillaries by a syringe pump (Harvard Apparatus, PUMP 11 ELITE, CA, USA)., respectively. The aqueous solution of 5 wt% PVA and silicone oil were used as the continuous phase, respectively. The mixture of TPGDA, 30% *v*/*v* AA aqueous solution (TAA), ethanol, and urea was used as the disperse phase. As the AA content increased, the number of phase separation steps decreased but the affinity of urea increased [18]. The volume fraction of AA in an aqueous solution was optimized to 30% *v*/*v*, and the TAA was confirmed as one of the ternary phases by considering the effect of the AA content on the adsorption property and the number of phase separation steps. We added 2-Hydroxy-2-methylpropiophenone (2% *v*/*v*) to the mixture as the photoinitiator and rhodamine B as the fluorescence agent. The phase separation process of a single emulsion droplet was captured with an optical microscope (Olympus, BX53, Sapporo, Japan) in a Petri dish. The ternary phase diagram could be made based on the mutual solubility of the mixed solution.

### 2.3. Preparation of the MISP

Microspheres with different structures (single-layer, double-layer, three-layer, and four-layer) could be obtained using UV light to irradiate the multi-emulsion droplets of TAA/ethanol/TPGDA/urea at special times in the phase separation process. After the microspheres were washed 5 times using sufficient PBS solution to remove the imprinting molecule (urea), the MISP was prepared. Meanwhile, the microspheres were prepared by droplets without urea (NISP).

### 2.4. Characterization of MISP

#### 2.4.1. Microscopic Analysis

To study the internal structure as well as the nanopore distribution of microspheres formed from different types of droplets, the cross-sections of microspheres with different structures were characterized using scanning electron microscopy (SEM, SU-8010, HITACHI, Ibaraki, Japan) at 5 kV.

#### 2.4.2. Swelling Property

The prepared MISP was naturally dried at room temperature for 24 h and immersed in water for 2 h. The images before and after swelling were recorded by microscopy, and the size changes of the microspheres were analyzed by Image J software (version 1.8.0).

(1) $$\mathrm{Swelling}\;\mathrm{ratio}\left(\%\right)=\frac{V_{s}}{V_{d}}$$

where *V*_s_ and *V*_d_ were the volumes of microspheres after and before swelling.

#### 2.4.3. Water Absorption

The dried MISP was weighed, and immersed in water for 2 h. Then, the water on the surface was absorbed using absorbent paper and weighed again. The water absorption property of MISP could be calculated from the equation as follows:

(2) $$\mathrm{Water}\;\mathrm{absorption}\;\mathrm{ratio}\;\left(\%\right)=\frac{W_{s}-W_{d}}{W_{d}}$$

where *W*_d_ and *W*_s_ were the weights of MISP before and after water absorption.

#### 2.4.4. IR Spectral Analysis

Attenuated Total Reflectance (ATR)–FTIR (Thermo Fisher, Thermo Scientific Nicolet 10, CLB, USA) was used to characterize the chemical structure of dried TPGDA, PAA, NISP (TPGDA/PAA), and MISP after urea adsorption (TPGDA/PAA+Urea), in which the PAA was tested in ATR mode, while the other samples were ground into powder and tested by the potassium bromide compression method.

#### 2.4.5. Thermal Stability

The TGA curves of dried TPGDA, PAA, TPGDA/PAA, and MISP after urea desorption (TPGDA/PAA-Urea) were examined using a thermal gravitational analysis apparatus (TA Instruments, TGA-Q500, New Castle, DE, USA). Approximately 10 mg of sample was heated from 50 °C to 600 °C at a rate of 10 °C/min in a nitrogen atmosphere.

#### 2.4.6. BET Analysis

After grinding MISP and NISP with three-layer structure into powder, 100 mg samples were analyzed by nitrogen adsorption-desorption using an N_2_ adsorption analyzer (Micromeritics, ASAP 2460, Norcross, GA, USA), respectively.

#### 2.4.7. Adsorption Study

To investigate the effect of MISP structure on the adsorption capacity, 40 mg of different structures of MISP (one-layer, two-layer, three-layer, and four-layer) were added to the 2 mL PBS solution of urea (3 mg/mL), respectively. After 24 h, 20 μL of the diluted solution was mixed with 80 μL of sulfuric acid solution (2 mol/L) and 200 μL of ethanol solution of DMAB (20 mg/mL). After the reaction for 5 min, the absorbance value at 440 nm was measured using a UV spectrophotometer (Agilent, Cary 100, Santa Clara, CA, USA). For the accuracy of the experimental results, when the tested urea concentration was greater than 1 mg/mL, all needed to be diluted three times before the DMAB reaction test. The standard curve of urea was y=0.1842x+0.0114, *R*^2^ = 0.9979, where *x* was the concentration of urea, and *y* was the absorbance value. By calculation, the adsorption capacity of MISP can be obtained. The next experiments were all conducted with the MISP with three-layer.

The dynamic adsorption capacity of MISP on urea can be tested as the following: 40 mg of MISP with three-layer were added to the 2 mL PBS solution of urea (3 mg/mL). At designed times, absorbance values of the supernatant were measured by the DMAB colorimetric method above. Then, NISP microspheres were tested the same as MISP above. The adsorption kinetic data of the microspheres were fitted by the following two adsorptions kinetic models [19].

(3) $$\mathrm{Pseudo}-\mathrm{first}-\mathrm{order}\;\mathrm{model}:\;Q_{t}=Q_{e}-Q_{e}\times \exp\left(-m_{1}t\right)$$

(4) $$\mathrm{Pseudo}-\mathrm{second}-\mathrm{order}\;\mathrm{model}:\;Q_{t}=\left(m_{2}Q_{e}{}^{2}t\right)/\left(1+m_{2}Q_{e}t\right)$$

where *Q*_e_ and *Q*_t_ were the adsorption capacity at equilibrium and at time t, respectively, while *m*_1_ and *m*_2_ were the rate constants corresponding to the pseudo-first-order and pseudo-second-order adsorption kinetic models, respectively. 

To test the isothermal adsorption properties of the MISP, 40 mg of samples were added to 2 mL PBS solution with different urea concentrations and immersed at room temperature for 24 h. After the adsorption reached equilibrium, the equilibrium adsorption capacity of the microspheres was examined by the DMAB colorimetric method described above. The equilibrium adsorption data were fitted using the Langmuir model and the Freundlich model, respectively. The expression of the Langmuir model is as follows:

*Q*_e_ = *Q*_max_*KC*_e_/(1 + *KC*_e_) (5)

where *C*_e_ is the concentration of the urea in the solution at equilibrium, *Q*_max_ is the maximum number of binding sites, and *K* is the binding association constant. The Freundlich model expression is as follows:

*Q*_e_ = *a C*_e_^*m*^ (6)

where *a* is a constant related to the binding capacity, and *m* represents the inhomogeneity index of the binding sites in the material.

We put 40 mg of MISP in 2 mL urea solution (3 mg/mL), tested the urea adsorption capacity after reaching adsorption equilibrium, then desorbed urea and retested the urea equilibrium adsorption capacity of the MISP. This process was repeated 4 times.

To study the adsorption of MISP for beneficial substances in dialysate, we tested their selective adsorption capacity using L-phenylalanine as a representative. Likewise, 40 mg of MISP were added to L-phenylalanine solutions with different concentrations. After 24 h reaching the adsorption equilibrium, the absorbance value of L-phenylalanine was measured at 257 nm using a UV spectrophotometer. The standard curve of L-phenylalanine was y=0.982x+0.1738, *R*^2^ = 0.996, where *x* was the concentration of L-phenylalanine, and *y* was the absorbance value. For the accuracy of the experiment, each group of experiments was repeated three times.

### 2.5. Fabrication of MISP-Embedded PDMS Chip

The PDMS chip was fabricated by standard photolithography and bonded using O_2_ plasma activation [20,21]. The width and height of the channel were designed to be 1000 μm and 245 μm, respectively. MISP with an average diameter of 220 μm was arranged in the channel by self-assembly and fixed with glutaraldehyde [22].

### 2.6. Numerical Simulation

Simulation analysis of the fluid flow inside the chip channel was conducted using COMSOL Multiphysics 5.5. According to the self-assembly arrangement regular of MISP in the chip, we designed the diameter of the microsphere model as 220 μm. The gaps between the microspheres were set as 2 μm based on the average value in the actual arrangement. The Navier–Stokes equation was used to describe the fluid flow in a channel as follows:

(7) $$\rho \frac{\partial u}{\partial t}+\rho \left(u\cdot \nabla \right)u=\nabla \cdot \left[-pI+\mu (\nabla u+{\left(\nabla u\right)}^{T}\right)]+F_{b}$$

where *ρ* (density of the fluid), *p* (viscosity of the fluid), and *μ* (pressure) were set based on the property of water, and *v* (velocity of the fluid) was set as 6.8 × 10^−3^ m/s, which could be converted to 1 mL/h.

## 3. Results and Discussions

### 3.1. The Phase Separation Process of the Ternary Mixture

**Figure 2:**
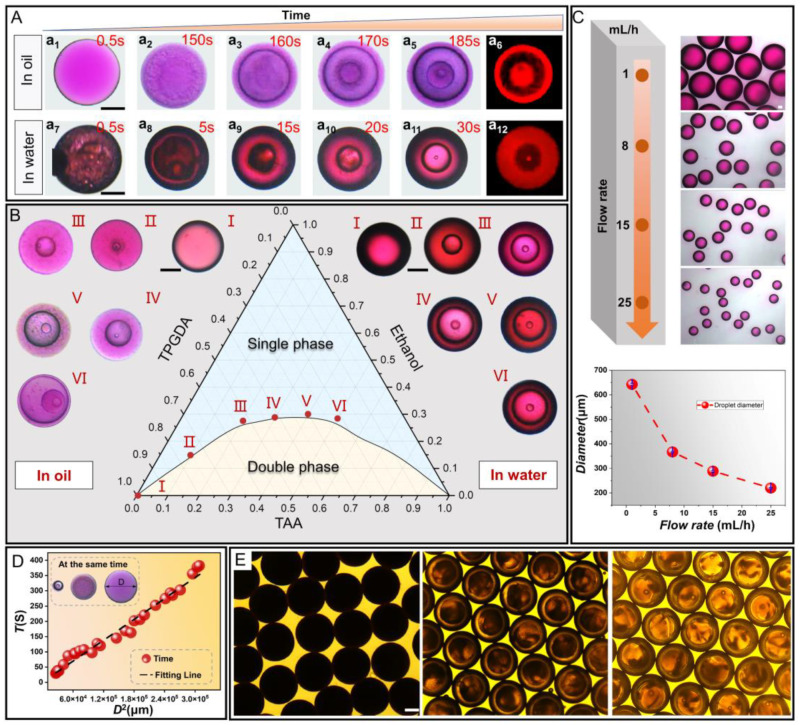
(**A**) The phase separation process of TPGDA/Ethanol/TAA droplets in silicon oil and PVA solution, respectively. (**B**) The multi-emulsion state of droplets in different compositions showed in the ternary phase diagram. (**C**) The effect of continuous phase flow rate (fixed disperse phase flow rate as 1 mL/h) on the size of prepared droplets in microfluidic systems and the corresponding diameter change curve. (**D**) The fit plots of initial droplet size versus squared phase separation time. (**E**) Microscopic images of one-layer, two-layer and three-layer microspheres after the LLPS droplets cured in PVA solution. All scale bars are 100 μm.

In a typical experiment, single emulsion droplets of TAA/Ethanol/TPGDA were prepared using a glass capillary microfluidic chip and immediately collected into Petri dishes for in situ observation. The phase separation states of the ternary mixed droplet were different based on the types of continuous phases. Thus, we studied the phase separation regulars in PVA aqueous solution (water) and silicone oil (oil) separately to select the most suitable parameters for urea adsorption. As shown in Figure 2A, the co-solubility balance between TPGDA and TAA was broken with the diffusion of ethanol in the droplet into the continuous phase. In the silicone oil, the TAA nanodroplets gradually separated from the original single emulsion droplet and formed a double emulsion droplet, as shown in Figure 2A(a_1_–a_3_). After that, TPGDA nanodroplets gradually separated from the core in that double emulsion droplet sequentially and formed a triple emulsion droplet (Figure 2A(a_4_)). Subsequently, the TAA riched in the third layer structure would precipitate as nanoparticles and aggregate into the fourth layer structure (Figure 2A(a_5_)). At this point, the phase separation process was completed. The TPGDA is an oil reagent that can be dyed by Rhodamine B, presenting red in the fluorescence microscope. The first and third layers of droplets showed the red state in Figure 2A(a_6_), which proved that the main component of the two layers was TPGDA. Similarly, the phase separation process of the ternary droplet in PVA aqueous solution was induced by the mass transfer of ethanol. Compared to silicone oil, the time to complete phase separation was greatly reduced as water has stronger solubility for ethanol, as shown in Figure 2A(a_7_–a_12_). We analyzed the phase separation process of the mixed droplets by the ternary phase diagram of TAA, ethanol, and TPGDA. As shown in Figure 2B, the binodal curve in the three-phase phase diagram could be obtained by observing the mutual solubility of the ternary liquids with different volume ratios. The multiplicity of droplets was determined by the number of consecutive steps of phase separation, where the composition of the innermost layer dictated whether the next step phase separation would occur. Thus, the initial composition is a key parameter for regulating droplet multiplicity. Increasing the content of ethanol and TAA along the binodal line could minimize the exchange of substances and maximize the number of the inner droplets [18]. In silicone oil, the single emulsion droplet without ethanol and TAA remained the same structure as in the initial preparation (Figure 2B(I)). When the ratio of TPGDA was reduced to 0.75 while the ratio of ethanol and TAA was increased to 0.1 and 0.15, respectively, the phase separation was induced by the diffusion of ethanol into the silicone oil, leading to the formation of the triple emulsion droplet. Due to the low content of the TAA, the sizes of the second and third layered droplets were relatively small at this point (Figure 2B(II)) and the phrase separation could not be continued in the third layer (TPGDA-riched layer). Likewise, by increasing the ratio of ethanol and TAA to 0.275, 0.2875, 0.3 and 0.2 (Figure 2B(III)), 0.3 (Figure 2B(IV)), 0.4 (Figure 2B(V)), respectively, we found that the droplets all had triple-layered structures after the phase separation was completed, which was different from the previous similar studies [23,24]. This phenomenon could attribute to the mutual solubility of AA and TPGDA. Therefore, the phase separation system requires a higher proportion of TAA to complete the next phase separation step. The increase of the fractions of ethanol and TAA to 0.2875 and 0.5 enabled to embrace more of the TAA components in the third layer of the droplet, which caused the formation of the fourth layer of the droplet (Figure 2B(VI)). Unlike in silicone oil, the phase separation of the ternary mixed droplet in PVA aqueous solution was induced by the diffusion of ethanol and the loss of TAA, which would lead to there being insufficient AA to form a quadruple emulsion droplet. The size of the initial droplets could be controlled by adjusting the flow rate ratio between the continuous and dispersed phases in a glass capillary microfluidic chip, as shown in Figure 2C.

The size of the droplet is a crucial factor in experimentally controlling the start time of the phase separation. As shown in Figure 2D, droplets with different sizes of the same components have different phase separation states at the same time. Droplets with sizes ranging from about 220 to 550 μm were found to be size-dependent in terms of phase separation start time. We found a significant linear relationship between the square of the droplet diameter *D*^2^ and the phase separation start time *T* by fitting the data, in which the fitting line had a slope of 0.0011. This relationship could be theoretically explained by the “Diameter-Square-Law” of droplet evaporation for the solution. That is, the time for droplet evaporation is proportional to the square of the droplet diameter [25]. The phase separation time could control the multiplicity of the droplet. After exposing droplets to UV light, we could get microspheres with corresponding structures. As shown in Figure 2E, we could observe that the single-layered, double-layered, and triple-layered microspheres prepared in PVA solution maintained the structures consistent with the droplets. However, microspheres prepared in silicone oil were opaque and their internal structure could not be observed by microscopy. Thus, we used Scanning Electron Microscope (SEM) to characterize the different structures of microspheres systematically.

### 3.2. The Cross-Sectional Morphology of MISP

**Figure 3:**
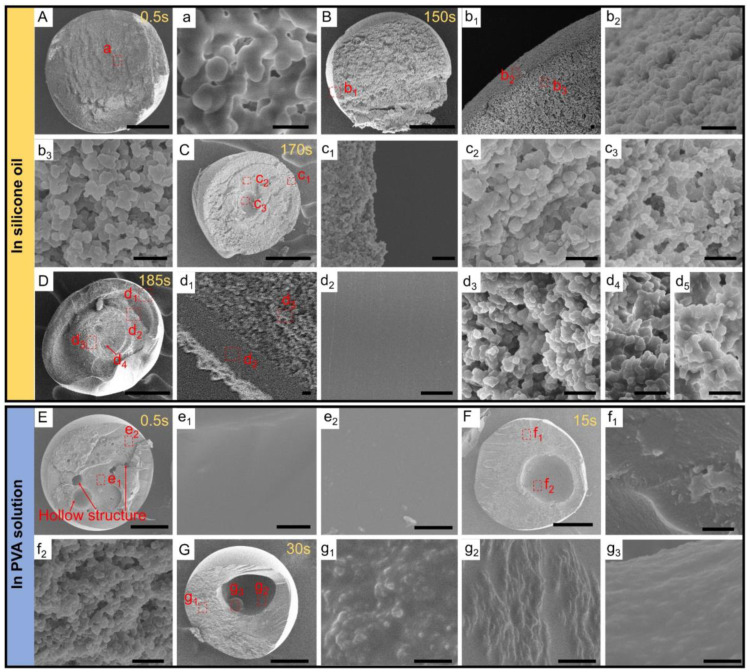
SEM images of the cross-sections of multi-layered MISP cured in silicone oil (**A**–**D**) and PVA solution (**E**–**G**) corresponding to the droplets at special times in Figure 2A. SEM images in (**a**,**b_1_**–**b_3_**,**c_1_**–**c_3_**,**d_1_**–**d_5_**,**e_1_**,**e_2_**,**f_1_**,**f_2_**,**g_1_**–**g_3_**) are the enlarged morphologies in (**A**–**G**) respectively, and the enlarged areas are marked with red dotted boxes in the corresponding figures. Scale bars in (**A**–**G**) are 200 μm and 1 μm in **a**–**g**.

Corresponding to the droplets in Figure 2A that had different phase separation states in silicone oil and PVA solution, we investigated the micro/nanostructure inside microspheres formed by droplets after UV exposure. At the initial single emulsion stage before phase separation in the silicone oil (Figure 2A(a_1_)), the mutual solubility of the ternary solution was gradually broken due to the loss of ethanol, and the TAA started to precipitate as micro-nano droplets, which formed interconnected network structures inside the microspheres after solidification, as shown in Figure 3A(a). However, this was a slow process and the phase separation phenomenon could not be observed on a macroscopic scale. When the phase separation process began, massive nanodroplets separated from the ternary droplet and formed a shell composed of TPGDA nanoparticles at the outermost layer of the microspheres, while the inner layer formed loose nanopores, as shown in Figure 3B(b_1_–b_3_). In the microspheres with three-layered structures, the distribution of TPGDA nanoparticles in the outermost layer (Figure 3C(c_1_)) compared with that at the beginning of phase separation (Figure 3B(b_2_)) was relatively loose. The inner layers were composed of nanoparticles and formed nanopores. The outermost layer of quadruple emulsion droplets in the bright field was the TPGDA-riched phase (Figure 2A(a_5_)), which was actually composed of two layers after solidification, as shown in Figure 3D(d_1_). In these two layers, most of the ethanol and TAA separated from the TPGDA, leading to the formation of the dense and smooth structure as the outermost layer, in which no nanopores could be observed, as shown in Figure 3D(d_2_). The other internal layers with nanopores were composed of nanoparticles. However, microspheres fabricated in PVA solution had different inner structures compared with that of silicone oil. Microspheres formed at the beginning of phase separation had hollow structures with different sizes inside (Figure 3E), which was due to the fast materials exchange in the PVA solution, decreasing the time of the phase separation process. The TAA would separate out as nanoparticles and aggregate into microdroplets of different sizes. However, due to the loss of AA to the PVA solution, the AA content of these droplets was too low to form PAA microspheres. Thus, the hollow structures were formed after washing and drying. For the same reason, the AA-riched phase in double and triple emulsion droplets could not form PAA after curing, as shown in Figure 3F,G. Besides, the internal structure was composed of dense and smooth TPGDA. Thus, MISP fabricated in PVA solution was not suitable for urea absorption. Depending on the above analysis, the outermost layer of the three-layered microspheres prepared in silicone oil had more nanopores compared with other microspheres, which was conducive to urea entering the interior of the microspheres to improve the adsorption property. By comparing the SEM images of cross-sections of MISP and NISP with the same structure, it was found that the presence of urea had little effect on the microstructure that could be observed (Figure S1). However, the results of Bet analysis of the same structure MISP and NISP showed that the parameters such as specific surface area and average pore size of MISP were larger than those of NISP, which indicated that the presence of urea template during the synthesis of MISP created cavities and effectively limited the shrinkage of pores during the polymerization process. So, the synthesized MISP had larger specific surface area and average pore size than NISP (Figure S2 and Table S1).

### 3.3. Properties of MISP

**Figure 4:**
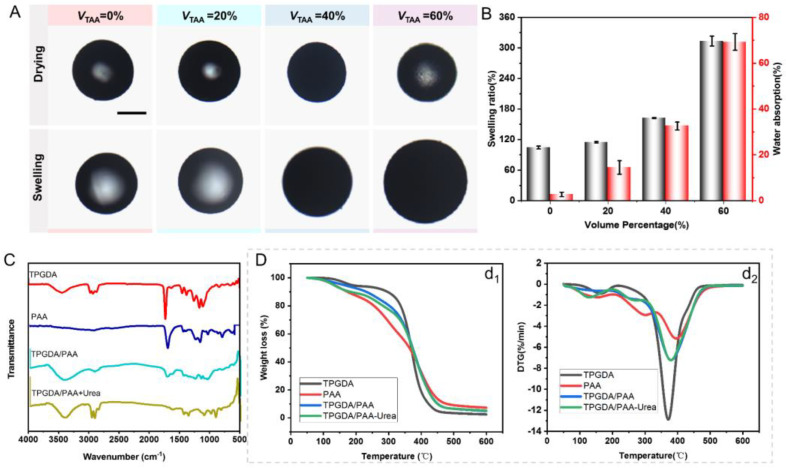
(**A**) Optical microscopic images of MISP made in with different TAA proportions before and after swelling. The scale bar is 200 μm. (**B**) The swelling and water absorption properties of MISP. (**C**) The infrared spectra of TPGDA, PAA, TPGDA/PAA (NISP), TPGDA/PAA + Urea (MISP after urea adsorption). (**D**) TG (**d_1_**) and DTG (**d_2_**) curves of TPGDA, PAA, TPGDA/PAA, TPGDA/PAA−Urea (MISP after urea desorption).

To study the reliability of microspheres as urea adsorbents, we characterized different properties of the microspheres. Swelling and water absorption are important properties of microspheres, which can reflect the exchange capacity of microspheres for urea in the dialysate. We found that the swelling property and water absorption capacity of microspheres were all endowed by the AA. When no AA was added to the microspheres (TPGDA microsphere), the swelling of the microspheres hardly occurred and the water absorption was also extremely low. However, the swelling ratio and water absorption of the microspheres gradually increased with the increase of the AA concentration, as shown in Figure 4A,B.

The asymmetric bending and shearing vibration of C-H in CH_3_ can be observed in 1435 cm^−1^, 1381 cm^−1^, respectively. The peak in 1110 cm^−1^ is the stretching vibration of C-O-C. The absorption peaks in the range of 842 cm^−1^~648 cm^−1^ are the out-of-plane bending vibration of C-H. The above characteristic absorption peaks are consistent with the typical characteristic structure of TPGDA [26]. In the infrared spectrum of PAA, the absorption peak at 2929 cm^−1^ is attributed to the CH_2_ vibration in the long chain of PAA. The sharp absorption peak at 1705 cm^−1^ is the C=O secondment vibration peak on the carboxyl group of the PAA molecular chain, while the absorption peaks around 1447 cm^−1^~1407 cm^−1^ correspond to the C-H bending vibration absorption peak of the PAA chain linkage. The absorption peaks at 1248 cm^−1^ and 1168 cm^−1^ are C-O stretching vibration absorption peaks [27]. Thus, the TPGDA and PAA were successfully fabricated. 

In the infrared spectrum of PAA/TPGDA, the absorption peak at 1724 cm^−1^ is a C=O stretching vibration in TPGDA/PAA, and the peak position is shifted to the high wavenumber compared to PAA (1705 cm^−1^) [28], indicating that there is a cross-linking reaction of TPGDA and PAA [29]. The weak absorption peak at 1646 cm^−1^ may be due to the small amount of C=C that was not fully polymerized. After absorbing urea, we could find new absorption peaks near 920 cm^−1^ in the infrared spectrum. The absorption peak could be attributed to the bending vibrations of N-H, indicating that the involvement of urea in the reaction makes the presence of N-H groups in the PAA/TPGDA molecular chain.

Figure 4D shows the thermostability of TPGDA, PAA, TPGDA/PAA, and TPGDA/PAA-Urea. TGA curve of TPGDA showed two distinct weight loss regions. The first degradation region was observed at 95–215 °C, which might be due to the degradation of the TPGDA monomer without cross-linking. The second degradation region was observed at 215–450 °C, which was due to the decomposition of cross-linked TPGDA [30]. TGA and DTG curves of PAA were consistent with previously reported studies [31]. The degradation region of TPGDA/PAA at 200–280 °C might be due to the decomposition of the carboxyl group of the PAA chain, and the degradation at 280–480 °C may be caused by the degradation of the backbone of TPGDA and PAA chains [32]. Compared to TPGDA/PAA, TPGDA/PAA-Urea had an increased weight loss region at about 90–190 °C, in which the weight of TPGDA/PAA-Urea lost only 3.47% more weight than TPGDA/PAA, and the weight degradation pattern in other temperature regions was approximately the same. Thus, the urea content adsorbed through the chemical chain was negligible [33].

### 3.4. Studies of Urea Removal of MISP

**Figure 5:**
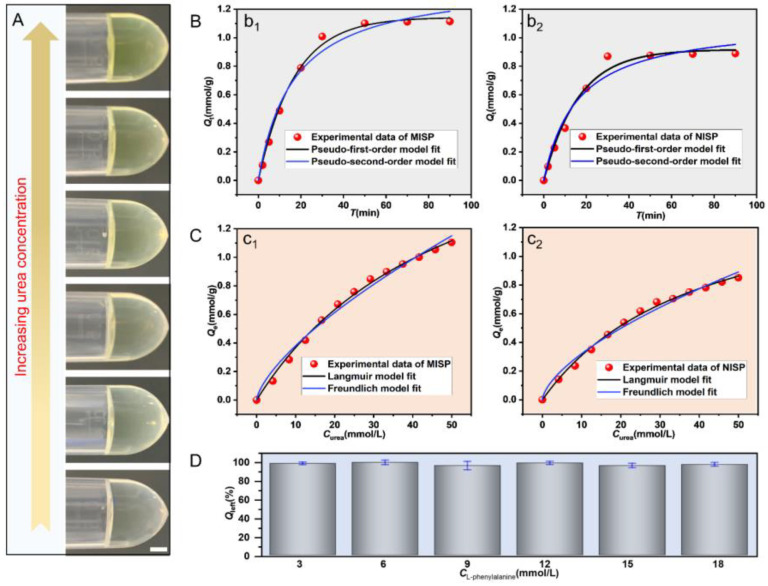
(**A**) Optical images of the reaction between DMAB with urea with the increase of urea concentration. The scale bar is 2 mm. (**B**) Average kinetic data and modeling for the binding of urea onto MISP (**b_1_**) and NISP (**b_2_**). (**C**) The equilibrium adsorption capacities of MISP (**c_1_**) and NISP(**c_2_**) for urea with the average data fitted by Langmuir and Freundlich models. (**D**) The adsorption capacities of MISP for L-phenylalanine.

DMAB can react with urea under acidic conditions to produce a lemon-yellow substance, and the color of the solution after reaction will be deep with the increase of urea concentration, as shown in Figure 5A. Thus, the urea concentration can be determined by the colorimetric method. Among the different types of MISP, the MISP with three-layer structure has a stronger urea adsorption capacity compared with others (Figure S3), so we chose that MISP for adsorption experiments. The adsorption of urea by MISP was higher than that of NISP microspheres at each time point, as shown in Figure 5B and Figure S4. This result indicated the successful construction of urea molecular imprinting sites. Besides, the adsorption process of urea on MISP could be divided into two phases: fast (the first 30 min) and slow. Of the equilibrium adsorption capacity, 88.5% was accomplished in the fast phase, which resulted from the presence of massive accessible sites. After 30 min of the adsorption process, the adsorption rate gradually slowed down and eventually reached equilibrium. The decrease of adsorption rate in the slow phase was mainly due to the decrease in residual urea concentration and the decrease in the number of accessible molecular imprinting sites.

In addition, we treated the adsorption kinetic data of MISP and NISP microspheres with pseudo-first-order and pseudo-second-order models, respectively. The fitted parameters of the corresponding two models and the correlation coefficients *R* are presented in Table S2. Comparing their correlation coefficients and combining the fitted curves, we found that the pseudo-first-order model (*R* > 0.99) was more suitable to describe the adsorption behavior of MISP. This also indicated that the diffusion of urea molecules inside the solution and microspheres controlled the whole adsorption process.

To investigate the isothermal adsorption performance of the microspheres, we tested the equilibrium adsorption amount of MISP at different initial concentrations of urea. As shown in Figure 5C and Figure S5, it could be found that along with the increase of the initial concentration of urea, the equilibrium adsorption capacity of both MISP and NISP increased accordingly. However, the adsorption amount of MISP was higher than that of NISP microspheres in the whole tested concentration range, further indicating the advantage of constructing the imprinted sites inside the material. Comparing the correlation coefficients *R* of the two models given in Supplementary Table S3, the Langmuir model could fit the experimental data better compared with the Freundlich model, which indicated that the binding sites inside the MISP were homogeneous. After the isothermal adsorption data of MISP and NISP were fitted by Equation (5), we calculated the maximum apparent number of binding sites for MISP and NISP microspheres as 2.268 mmol/g and 1.56 mmol/g, respectively (Table S3). To evaluate the reusability of the MISP, a series of adsorption–desorption experiments were performed. The adsorption–desorption experiments were conducted using the same kind of MISP for a total of four cycles. The equilibrium adsorption capacities of the regenerated MISP for different adsorption-desorption cycles are given in Figure S6. It can be seen that the adsorption capacity of MISP for urea decreased to some extent during the four adsorption–desorption cycles. This indicated that the regeneration process of MISP microspheres leads to the failure of some binding sites, but the amount of failure is limited. To investigate the adsorption capacity of MISP for beneficial substances in dialysate, we examined the isothermal adsorption property of the MISP on L-phenylalanine. The results showed that the MISP had almost no adsorption capacity for L-phenylalanine, indicating that the MISP had specificity for urea adsorption and would not cause loss of nutrients such as amino acids in the dialysate.

### 3.5. Urea Removal in MISP-Integrated Microfluid Chip

**Figure 6:**
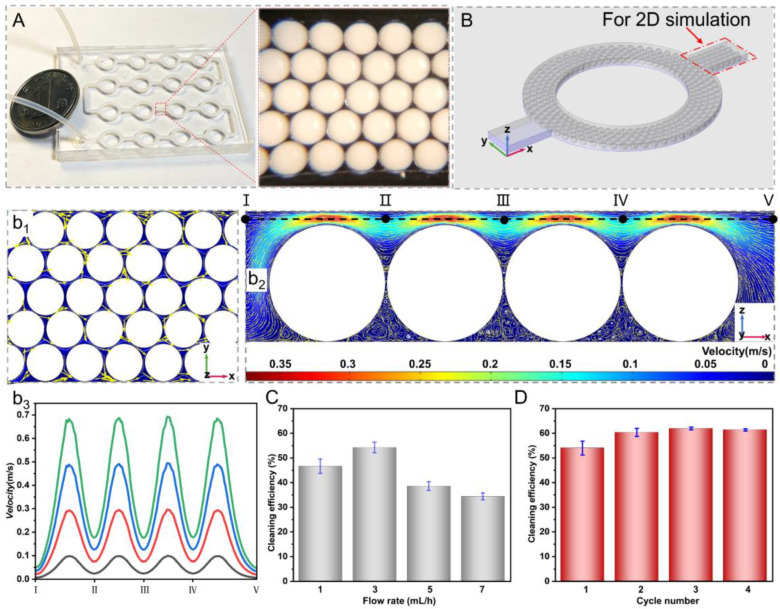
(**A**) Optical images of the MISP integrated microfluidic chip and the details of the MISP arrangement by self-assembly. (**B**) The numerical simulation of the fluid flowing in the vertical (z = 110 μm, **b_1_**) and horizontal directions (y = 110 μm, **b_2_**), and the cut-off velocity analysis (**b_3_**) corresponding to b_2_. Effect of flow rate (**C**) and cycle numbers (**D**) of adsorption in the chip on cleaning efficiency.

Considering that the dialysate was flowing in the WRK, we designed a circularly connected microfluidic channel and embedded the self-assembled MISP in the bottom of the channel by glutaraldehyde [22], which could generate turbulence of fluid to enhance the contact between urea and MISP (Figure 6A). We conducted the qualitative analysis of the fluid flow in the channel by simulation, as shown in Figure 6B. It was found that the fluid flow lines between the microspheres and the channel were distorted in different 2D planes (Figure 6B(b_1_,b_2_)). Besides, the velocity on the same cutoff varied greatly and increased with the flow rate, as shown in Figure 6B(b_3_). Thus, the design of the channel and microsphere arrangement could improve the mixing efficiency of urea. It is worth noting that the removal rate of urea in the microfluidic chip was related to the initial flow rate. With the increase of the flow rate, the adsorption of urea showed a characteristic of first increasing and then decreasing. This phenomenon might be explained as follows: the mixing of fluid with MISP was not very intense at low flow rates, but at high flow rates, a large amount of fluid flowed over the top of the channel, which likewise led to a decrease in the contact between the fluid and MISP. Therefore, we chose a velocity of 3 mL/h for cyclic adsorption experiments and the results showed that one cycle of adsorption could make the removal efficiency of urea reach about 54%, and the adsorption equilibrium of microspheres could be reached after three cycles of adsorption.

## 4. Conclusions

In conclusion, we proposed a new liquid–liquid phase separation reagent system consisting of TPGDA, ethanol, and TAA, and prepared urea molecular imprinted microspheres with nanopore and different structures based on this formula. By adjusting the ratios of the components, we could control the properties of the microspheres such as swelling, water absorption, and internal structure. In addition, the urea molecular imprinted microspheres could recognize and adsorb urea well and did not affect beneficial materials. Besides, the self-assembled arrangement of these microspheres in the microfluidic channel could enhance the contact between the fluid and the microspheres, which was beneficial to improving the adsorption efficiency. Therefore, the urea adsorption platform has great potential to serve as a component in the development of wearable artificial kidneys.

## Data Availability

The data presented in this study are available on request from the corresponding author. All of the raw data are not presented due to their type and large number.

## Supplementary Materials

The following supporting information can be downloaded at: https://www.mdpi.com/article/10.3390/nano12030418/s1, Figure S1: SEM images of the cross-sections of multi-layered NISP cured in silicone oil (A–C) corresponding to the droplets at special times in Figure 3A–C; Figure S2: N_2_ adsorption-desorption isotherms of MISP (A) and NISP (B). The inset is the pore size distribution.; Figure S3: The relationship between the structure of MISP and urea adsorption capacity; Figure S4: Kinetic data and for the binding of urea onto MISP and MISP.; Figure S5: The equilibrium adsorption data for the binding of urea onto MISP and MISP.; Figure S6: The urea adsorption capacity of MISP for repeated use; Table S1: BET parameters of the MISP and NISP; Table S2: Parameters of urea adsorption kinetics fitted by two models for MISP and NISP; Table S3: Binding isotherm constants of urea onto MISP and NISP.

## References

[1] Gao R., Yang B., Chen C., Chen F., Chen C., Zhao D., Lv X. (2021). Recognition of chronic renal failure based on Raman spectroscopy and convolutional neural network. Photodiagnosis Photodyn. Ther..

[2] Hao Y., Yuan X., Yan J., Pham M., Rohlsen D., Qian P., Cheng F., Wang Y. (2019). Metabolomic Markers in Tongue-Coating Samples from Damp Phlegm Pattern Patients of Coronary Heart Disease and Chronic Renal Failure. Dis. Markers.

[3] Bikbov B., Purcell C.A. (2020). Global, regional, and national burden of chronic kidney disease, 1990–2017: A systematic analysis for the Global Burden of Disease Study 2017. Lancet.

[4] Pittard J.D., Nissenson A.R., Fine R.N. (2017). Safety Monitors in Hemodialysis. Handbook of Dialysis Therapy.

[5] Davenport A., Gura V., Ronco C., Beizai M., Ezon C., Rambod E. (2007). A wearable haemodialysis device for patients with end-stage renal failure: A pilot study. Lancet.

[6] Gura V., Macy A.S., Beizai M., Ezon C., Golper T.A. (2009). Technical Breakthroughs in the Wearable Artificial Kidney (WAK). Clin. J. Am. Soc. Nephrol..

[7] Cheah W.-K., Sim Y.-L., Yeoh F.-Y. (2016). Amine-functionalized mesoporous silica for urea adsorption. Mater. Chem. Phys..

[8] Kim J.C., Ronco C. (2011). Current technological approaches for a wearable artificial kidney. Contrib. Nephrol..

[9] Lehmann H.D., Marten R., Gullberg C.A. (1981). How To Catch Urea? Considerations on Urea Removal from Hemofiltrate. Artif. Organs.

[10] Wester M., van Gelder M.K., Joles J.A., Simonis F., Hazenbrink D.H.M., van Berkel T.W.M., Vaessen K.R.D., Boer W.H., Verhaar M.C., Gerritsen K.G.F. (2018). Removal of urea by electro-oxidation in a miniature dialysis device: A study in awake goats. Am. J. Physiol. Renal. Physiol..

[11] Abidin M.N.Z., Goh P.S., Ismail A.F., Said N., Othman M.H.D., Hasbullah H., Abdullah M.S., Ng B.C., Kadir S.H.S.A., Kamal F. (2018). Highly adsorptive oxidized starch nanoparticles for efficient urea removal. Carbohydr. Polym..

[12] Kameda T., Horikoshi K., Kumagai S., Saito Y., Yoshioka T. (2020). Adsorption of urea, creatinine, and uric acid onto spherical activated carbon. Sep. Purif. Technol..

[13] Wilson L.D., Xue C. (2013). Macromolecular sorbent materials for urea capture. Appl. Polyer.

[14] Charrier E.E., Pogoda K., Wells R.G., Janmey P.A. (2018). Control of cell morphology and differentiation by substrates with independently tunable elasticity and viscous dissipation. Nat. Commun..

[15] Ferreira L., Vidal M.M., Gil M.H. (2000). Evaluation of poly (2-hydroxyethyl methacrylate) gels as drug delivery systems at different pH values. Int. J. Pharm..

[16] Calixto G., Yoshii A.C., Rocha e Silva H., Stringhetti Ferreira Cury B., Chorilli M. (2015). Polyacrylic acid polymers hydrogels intended to topical drug delivery: Preparation and characterization. Pharm. Dev. Technol..

[17] Zhang S., Wang X., Man J., Li J., Cui X., Zhang C., Shi W., Li D., Zhang S., Li J. (2020). Histone Deacetylase Inhibitor-loaded Calcium Alginate Microspheres for Acute Kidney Injury Treatment. ACS Appl. Bio Mater..

[18] Park S., Lee S.S., Kim S.-H. (2020). Photonic Multishells Composed of Cholesteric Liquid Crystals Designed by Controlled Phase Separation in Emulsion Drops. Adv. Mater..

[19] Wang Z., Qiu T., Guo L., Ye J., He L., Li X. (2018). Polymerization induced shaping of Pickering emulsion droplets: From simple hollow microspheres to molecularly imprinted multicore microrattles. Chem. Eng. J..

[20] Sun H., Ren Y., Tao Y., Jiang T., Jiang H. (2020). Three-Fluid Sequential Micromixing-Assisted Nanoparticle Synthesis Utilizing Alternating Current Electrothermal Flow. Ind. Eng. Chem. Res..

[21] Chen X., Ren Y., Liu W., Feng X., Jia Y., Tao Y., Jiang H. (2017). A Simplified Microfluidic Device for Particle Separation with Two Consecutive Steps: Induced Charge Electro-osmotic Prefocusing and Dielectrophoretic Separation. Anal. Chem..

[22] Fan J.-B., Luo J., Luo Z., Song Y., Wang Z., Meng J., Wang B., Zhang S., Zheng Z., Chen X. (2019). Bioinspired Microfluidic Device by Integrating a Porous Membrane and Heterostructured Nanoporous Particles for Biomolecule Cleaning. ACS Nano.

[23] Haase M.F., Brujic J.J.A.C. (2015). Tailoring of High-Order Multiple Emulsions by the Liquid–Liquid Phase Separation of Ternary Mixtures. Angew. Chem..

[24] Liang S., Li J., Man J., Chen H.J.L. (2016). Mass-Transfer-Induced Multistep Phase Separation in Emulsion Droplets: Toward Self-Assembly Multilayered Emulsions and Onionlike Microspheres. Langmuir.

[25] Jakubczyk D., Kolwas M., Derkachov G., Kolwas K., Zientara M. (2012). Evaporation of Micro-Droplets: The “Radius-Square-Law Revisited”. Acta Phys. Pol. A.

[26] Shirajuddin S.S.M., Ratnam C.T., Hussin K., Shukri N.A., Ishak N.S. (2020). Quantification of Tripropylene Glycol Diacrylate grafted onto Waste Tire Dust from Fourier Transform Infrared Spectroscopy. Mater. Today Proc..

[27] Yi X., Xu Z., Liu Y., Guo X., Ou M., Xu X. (2017). Highly efficient removal of ur’’anium(vi) from wastewater by polyacrylic acid hydrogels. RSC Adv..

[28] Singh R., Pal D., Mathur A., Singh A., Krishnan M.A., Chattopadhyay S. (2019). An efficient pH sensitive hydrogel, with biocompatibility and high reusability for removal of methylene blue dye from aqueous solution. React. Funct. Polym..

[29] Lewis P.C., Graham R.R., Nie Z., Xu S., Seo M., Kumacheva E. (2005). Continuous Synthesis of Copolymer Particles in Microfluidic Reactors. Macromolecules.

[30] Abid C.K.V.Z., Jain S., Jackeray R., Chattopadhyay S., Singh H. (2017). Formulation and characterization of antimicrobial quaternary ammonium dendrimer in poly(methyl methcarylate) bone cement. J. Biomed. Mater. Res. Part. B.

[31] Chen Q., Yu H., Wang L., Abdin Z.-u., Yang X., Wang J., Zhou W., Zhang H., Chen X. (2016). Synthesis and characterization of amylose grafted poly(acrylic acid) and its application in ammonia adsorption. Carbohydr. Polym..

[32] Witono J.R., Marsman J.H., Noordergraaf I.-W., Heeres H.J., Janssen L.P.B.M. (2013). Improved homopolymer separation to enable the application of 1H NMR and HPLC for the determination of the reaction parameters of the graft copolymerization of acrylic acid onto starch. Carbohydr. Res..

[33] Chen H., Bian F., Sun L., Zhang D., Shang L., Zhao Y. (2020). Hierarchically Molecular Imprinted Porous Particles for Biomimetic Kidney Cleaning. Adv. Mater..

