# Supplementary material for: Controllable Fabrication of Molecularly Imprinted Microspheres with Nanoporous and Multilayered Structure for Dialysate Regeneration
Source: Nanomaterials (Basel). 2022 Jan 27;12(3):418. doi: 10.3390/nano12030418 (PMC8840109; doi:10.3390/nano12030418)
Supplement: Supplementary file 1 [file nanomaterials-12-00418-s001.zip › nanomaterials-1522767-supplementary.pdf]

Supplementary Materials

Controllable Fabrication of Molecularly Imprinted Microspheres with Nanoporous and Multilayered Structure for Dialysate Regeneration

Hongchi Wu [1,*], Shanguo Zhang [2], Lu Liu [1], Yukun Ren [2,3], Chun Xue [1], Wenlong Wu [2], Xiaoming Chen [4] and Hongyuan Jiang [2,*]

[1] Department of Nephrology, First Affiliated Hospital of Harbin Medical University, 23 Youzheng Street, Harbin 150001, China; 2020020649@hrbmu.edu.cn (L.L.); 2021020689@hrbmu.edu.cn (C.X.)
[2] School of Mechatronics Engineering, Harbin Institute of Technology, West Da-zhi Street 92, Harbin 150001, China; 21b908081@stu.hit.edu.cn (S.Z.); rykhit@hit.edu.cn (Y.R.); hit_wu@stu.hit.edu.cn (W.W.)
[3] State Key Laboratory of Robotics and System, Harbin Institute of Technology, West Da-zhi Street 92, Harbin 150001, China
[4] School of Control Engineering, Northeastern University at Qinhuangdao, Qinhuangdao 066004, China; chenxiaoming@neuq.edu.cn
* Correspondence: wuhc@hrbmu.edu.cn (H.W.); jhy_hit@hit.edu.cn (H.J.)

Citation: Wu, H.; Zhang, S.; Liu, L.; Ren, Y.; Xue, C.; Wu, W.; Chen, X. Controllable Fabrication of Molecularly Imprinted Microspheres with Nanoporous and Multilayered Structure for Dialysate Regeneration. *Nanomaterials* **2022**, *12*, 418. https://doi.org/10.3390/nano12030418

Academic Editors: Nunzio Denora and Ilaria Arduino

Received: 8 December 2021
Accepted: 25 January 2022
Published: 27 January 2022

Publisher's Note: MDPI stays neutral with regard to jurisdictional claims in published maps and institutional affiliations.

Copyright: © 2022 by the authors. Licensee MDPI, Basel, Switzerland. This article is an open access article distributed under the terms and conditions of the Creative Commons Attribution (CC BY) license (https://creativecommons.org/licenses/by/4.0/).

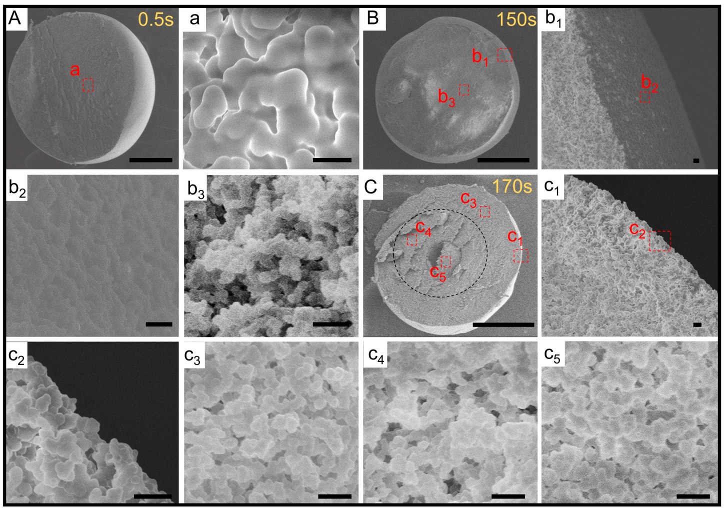

Figure S1. SEM images of the cross-sections of multi-layered NISP cured in silicone oil (**A**–**C**) corresponding to the droplets at special times in Figure 3**A**–**C**. The enlarged morphologies of each layer (a–c). Scale bars in **A**–**C** are 200µm and 1µm in a–c.

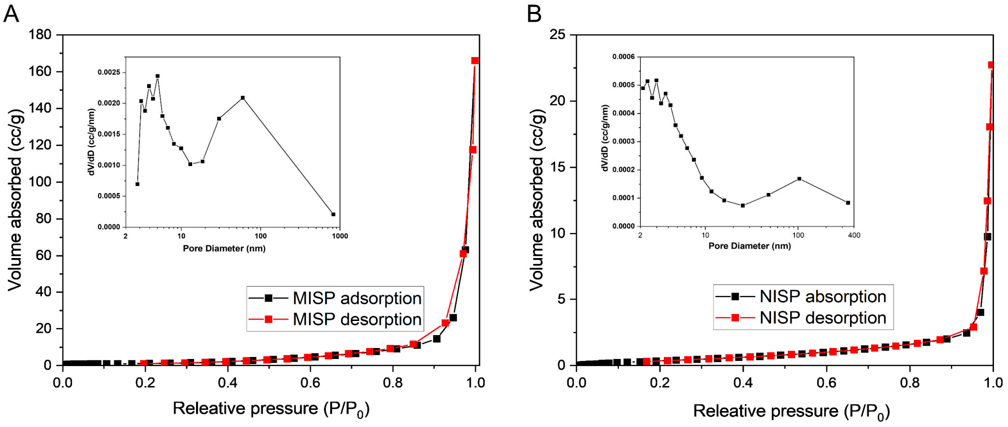

Figure S2. N_2 adsorption-desorption isotherms of MISP (**A**) and NISP (**B**). The inset is the pore size distributions.

Table S1. BET parameters of the MISP and NISP.

Properties	MISP	NISP
Surface area (m^2 g^{-1})	1.2709	4.0183
Average pore radius (nm)	55.35	171.81

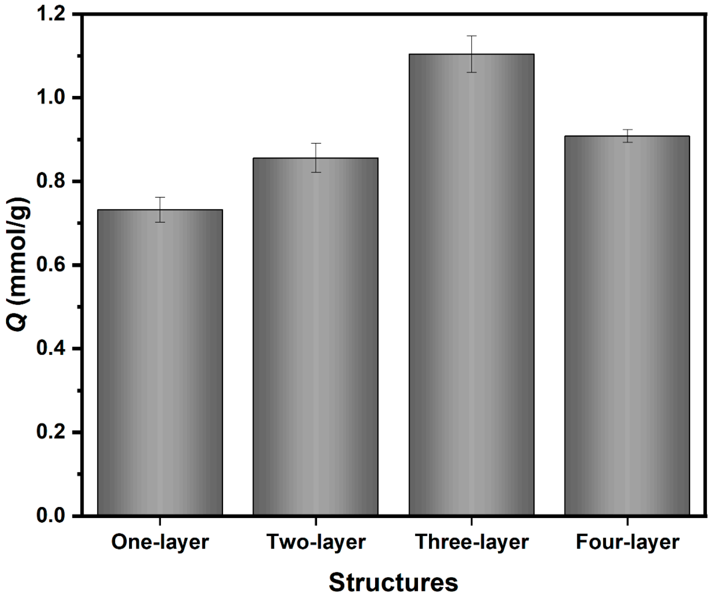

Figure S3. The relationship between the structure of MISP and urea adsorption capacity.

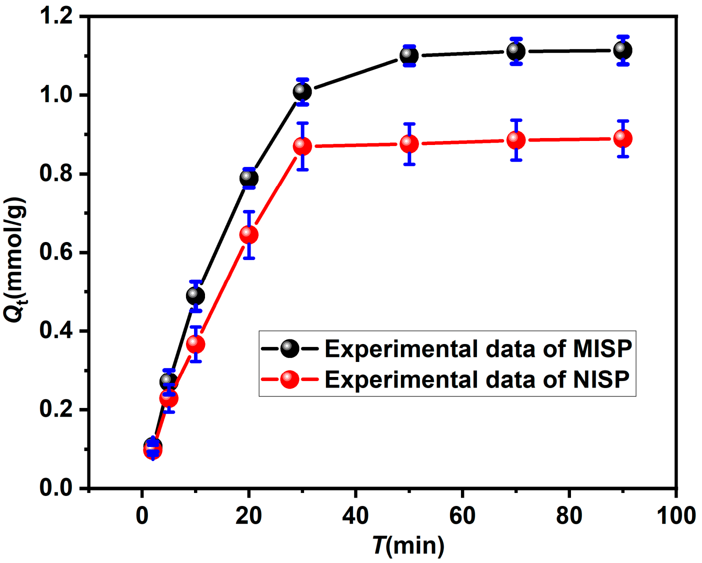

Figure S4. Kinetic data and for the binding of urea onto MISP and MISP.

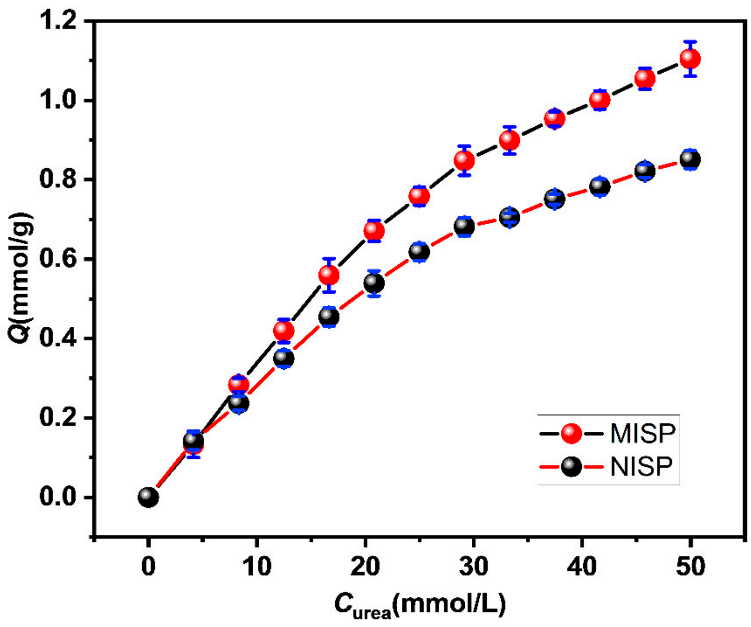

Figure S5. The equilibrium adsorption data for the binding of urea onto MISP and MISP.

Table S2. Parameters of urea adsorption kinetics fitted by two models for MISP and NISP.

Sample	Q_e'(mmol/g)	Pseudo-first-order model			Pseudo-second-order model		
		R^2	Q_e''(mmol/g)	m_1(min^{-1})	R^2	Q_e'' (mmol/g)	m_2(min^{-1})
**MISP**	1.114	0.9959	1.143	0.059	0.9823	1.401	0.043
NISP	0.889	0.9856	0.917	0.062	0.9671	1.119	0.057

Q_e' is the experimental value of Q_e. .

Q_e'' is the calculated value of Q_e.

m_1 is the rate constant of first-order adsorption.

m_2 is the rate constant of second-order adsorption.

Table S3. Binding isotherm constants of urea onto MISP and NISP.

Sample	Langmuir model			Freundlich model		
	R^2	Q_{max}(mmol/g)	K	R^2	a	m
MISP	0.9951	2.268	0.322	0.9862	0.078	0.687
NISP	0.9948	1.56	0.025	0.9853	0.074	0.635

Q_{max} is the apparent maximum number of binding sites, K is the binding association constant, and R^2 is the correlation coefficient value.

a is the Freundlich constant, m is the heterogeneity index.

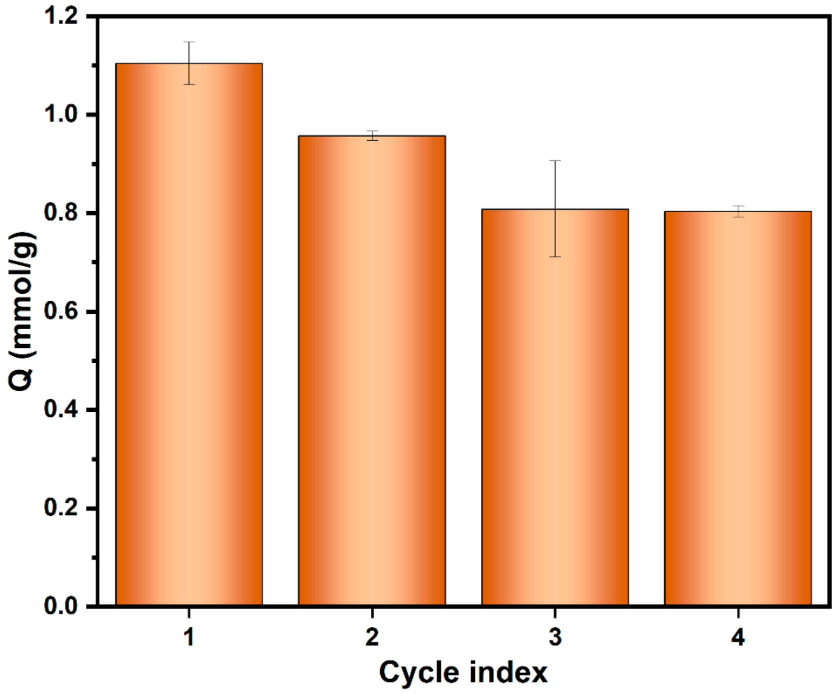

Figure S6. The urea adsorption capacity of MISP for repeated use.
